# Beneficial Renal and Pancreatic Phenotypes in a Mouse Deficient in FXYD2 Regulatory Subunit of Na,K-ATPase

**DOI:** 10.3389/fphys.2016.00088

**Published:** 2016-03-07

**Authors:** Elena Arystarkhova

**Affiliations:** Laboratory of Membrane Biology, Neurosurgery, Massachusetts General HospitalBoston, MA, USA

**Keywords:** gamma subunit, knockout mouse, renal sodium transporters, hypertension, adaptation, pancreatic islets, beta cells

## Abstract

The fundamental role of Na,K-ATPase in eukaryotic cells calls for complex and efficient regulation of its activity. Besides alterations in gene expression and trafficking, kinetic properties of the pump are modulated by reversible association with single span membrane proteins, the FXYDs. Seven members of the family are expressed in a tissue-specific manner, affecting pump kinetics in all possible permutations. This mini-review focuses on functional properties of FXYD2 studied in transfected cells, and on noteworthy and unexpected phenotypes discovered in a *Fxyd2*^−∕−^ mouse. FXYD2, the gamma subunit, reduces activity of Na,K-ATPase either by decreasing affinity for Na^+^, or reducing V_max_. FXYD2 mRNA splicing and editing provide another layer for regulation of Na,K-ATPase. In kidney of knockouts, there was elevated activity for Na,K-ATPase and for NCC and NKCC2 apical sodium transporters. That should lead to sodium retention and hypertension, however, the mice were in sodium balance and normotensive. Adult *Fxyd2*^−∕−^ mice also exhibited a mild pancreatic phenotype with enhanced glucose tolerance, elevation of circulating insulin, but no insulin resistance. There was an increase in beta cell proliferation and beta cell mass that correlated with activation of the PI3K-Akt pathway. The *Fxyd2*^−∕−^ mice are thus in a highly desirable state: the animals are resistant to Na^+^ retention, and showed improved glucose control, i.e., they display favorable metabolic adaptations to protect against development of salt-sensitive hypertension and diabetes. Investigation of the mechanisms of these adaptations in the mouse has the potential to unveil a novel therapeutic FXYD2-dependent strategy.

## Regulatory subunits of Na,K-ATPase

**Figure 1:**
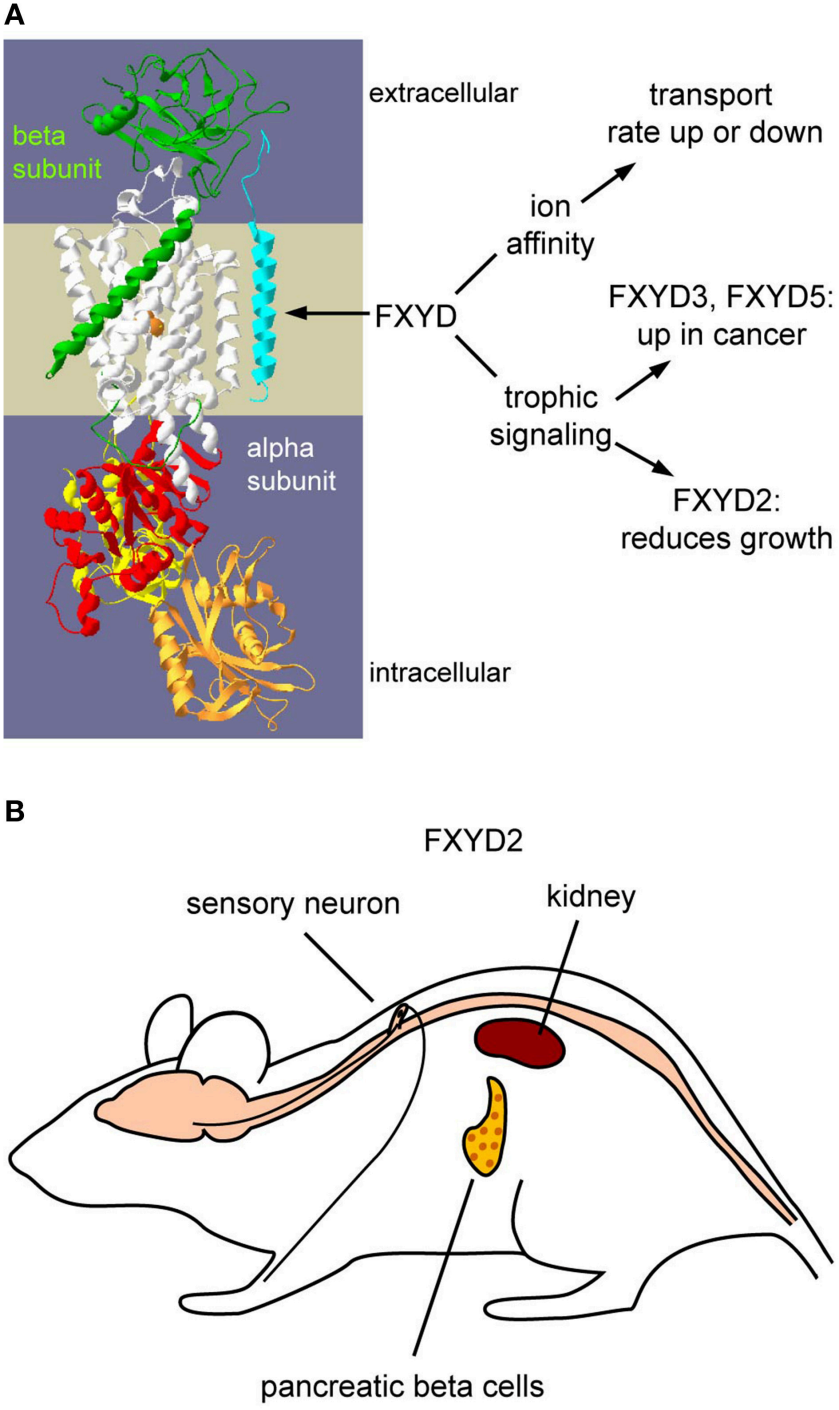
**Functions of FXYDs and the tissue distribution of FXYD2. (A)** Schematic representation of how FXYD proteins alter functions of Na,K-ATPase. Effects on affinities for substrates are well-characterized. How FXYD proteins function at the molecular level for the demonstrated alterations of signaling pathways has yet to be determined. **(B)** Expression sites of FXYD2 in rodents. The principal sites are renal tubules, pancreatic beta cells, and sensory neurons. Mammary glands may also express FXYD2, but this remains to be systematically investigated.

FXYDs are established regulators of Na,K-ATPase (Garty and Karlish, 2005; Geering, 2006). This is a family of seven small single span membrane proteins (FXYD1-FXYD7) with differential and actively regulated expression in tissues and cells (Sweadner and Rael, 2000; Geering, 2006; Figure 1A). Association of each with Na,K-ATPase leads to modulation of activity of the enzyme in all possible ways: inhibition or activation of V_max_, and increase or decrease of affinity for Na^+^, K^+^, or ATP. A remarkable feature of FXYD proteins is their interchangeability, which allows fine-tuning of kinetic properties of the Na,K-ATPase in order to adjust to any given physiological or pathological situation (Arystarkhova et al., 2007). Based on crystal structures of Na,K-ATPase, the FXYD subunit is positioned on the periphery of the complex (Toyoshima et al., 2011), which may be a structural basis for “easy” exchange of regulatory subunits. Association of FXYD proteins with alpha/beta complex occurs post-translationally either in Golgi or even at the plasma membrane (Crambert et al., 2002; Pihakaski-Maunsbach et al., 2008; Moshitzky et al., 2012). This may facilitate exchange of regulatory subunits within an already-functioning complex.

FXYD2 protein was the first member of the family associated with Na,K-ATPase: it was identified as a proteolipid labeled with a photosensitive-derivative of ouabain, specific inhibitor of Na,K-ATPase, along with the alpha and beta subunits (Forbush et al., 1978). Further analysis of the “gamma subunit” (FXYD2) confirmed its intimate relation to Na,K-ATPase by co-localization and co-immunoprecipitation with the alpha subunit from kidney membranes (Mercer et al., 1993). Distribution of FXYD2 (at least in rodent) is quite limited: it is highly expressed in kidney with lower representation in pancreas and even lower in mammary gland (Sweadner et al., 2000) and dorsal root ganglia (Venteo et al., 2012; Wang et al., 2015; Figure 1B). FXYD2 stands out from the family by being present in two splice variants: FXYD2a and FXYD2b, that differ only at the small N-terminal segment exposed on the extracellular side (Küster et al., 2000; Sweadner et al., 2000; Arystarkhova et al., 2002b). The distribution patterns of the FXYD2 splice variants in kidney are not identical (Pu et al., 2001; Arystarkhova et al., 2002b). Co-expression of FXYD2a and FXYD2b is seen in medullary thick ascending limb. Proximal convoluted tubules however, express only FXYD2a, while only FXYD2b is found in distal convoluted tubules and connecting tubules. The data suggest engagement of the distinct segments of FXYD2a and FXYD2b in extracellular association with tubule-specific partners that are not identified yet. It should be noted that association with multiple partners has been described for other FXYD proteins, such as FXYD1 with Na^+^/Ca^2+^ exchanger (Wang et al., 2006) or L-type Ca^2+^ channel (Guo et al., 2010), and FXYD4 with KCNQ1 K^+^ channel (Jespersen et al., 2006).

FXYDs can also influence cell growth through downstream signaling pathways. FXYD3 and FXYD5 (dysadherin) are expressed in some normal tissues but are elevated in some types of cancer (Arimochi et al., 2007; Nam et al., 2007). We have demonstrated that FXYD2 decreases cell growth rates in culture (Arystarkhova et al., 1999, 2002a, 2007; Wetzel et al., 2004), while FXYD3 has been demonstrated to increase growth rates (Kayed et al., 2006). This is yet another example of the multiplicity of ways that different FXYDs can act.

## Functional properties of FXYD2 learned from transfected cells

Remarkably, no endogenously expressed FXYD2 protein is found in any renal mammalian cell line (Arystarkhova et al., 2007); this is also true of a pancreatic beta cell line (Arystarkhova et al., 2013). Functional assessment of FXYD2 has been explored in expression systems. Reduction of the affinity for Na^+^ is the most widely-supported effect of FXYD2 (Béguin et al., 1997; Arystarkhova et al., 1999; Therien et al., 1999). Notably, both splice variants, FXYD2a and FXYD2b, similarly inhibited Na,K-ATPase by decreasing apparent affinity for Na^+^ (Arystarkhova et al., 2002a). This rules out the N-terminal splice variants for modulation of this kinetic parameter. Further structure-function studies implicated the transmembrane segment of FXYD proteins in the control of Na^+^ affinity (Lindzen et al., 2003).

Unexpectedly though, an unidentified post-translational modification relieved FXYD2a-mediated reduction of apparent Na^+^ affinity in stably-transfected cells (Arystarkhova et al., 2002a). Post-translational modification is a common theme in the FXYD family: phosphorylation (Silverman et al., 2005; Fuller et al., 2009), palmitoylation (Tulloch et al., 2011), *O*-glycosylation (Tsuiji et al., 2003; Crambert et al., 2004), and *S*-glutathionylation (Bibert et al., 2011) have all been reported for various FXYDs. The palmitoylation and *S*-glutathionylation entail modification of one or both cysteine residues immediately following the transmembrane span. Palmitoylation was required for Na,K-ATPase inhibition by FXYD1 (Tulloch et al., 2011), while *S*-glutathionylation of FXYD1 correlated with reduction of inhibition mediated by *S*-glutathionylation of the β1 subunit (Bibert et al., 2011). FXYD2 is not susceptible to *S*-glutathionylation (Bibert et al., 2011), but in silico analysis of FXYD2a structure predicts Cys52 (FXYD2a numbering) as a potential site for palmitoylation (Tulloch et al., 2011). However, whether FXYD2 is indeed palmitoylated and whether this kind of regulation takes place *in vivo* awaits further investigation.

In addition to mRNA splicing and post-translational modifications, we observed that mRNA for FXYD2b could be selectively edited, at least in transfected cells (Sweadner et al., 2011). Single base substitution C172U of the RNA results in introduction of a premature in-frame stop codon. The truncated protein was retained intracellularly due to exposure of an ER retrieval signal, -KKXX. The RNA editing was observed in several established cell lines of different origin (NRK-52E renal epithelial cells, C6 glioma, and L6 myotubes) thus generalizing the phenomenon. Functionally, expression of the truncated form of FXYD2b completely abrogated the reduction of apparent Na^+^ affinity of Na,K-ATPase activity. Cells expressing truncated FXYD2b also exhibited no growth delay as opposed to transfectants with full length FXYD2b. Remarkably, no truncation was found for FXYD2a, suggesting selective post-transcriptional control of FXYD2b. This is a potential indirect mechanism for differential regulation of Na^+^ affinity by the splice forms. Whether the modification of the transcript plays a protective role, ensuring that FXYD2b is inactive even if made in cells where it may be deleterious, or the truncated protein has another biological role, remains to be discovered.

FXYD2a was upregulated in cells by several types of cellular stress, including hyperosmotic and oxidative (Wetzel et al., 2004). In these conditions it reduced the V_max_ of the enzyme. The stress-related response was observed in cells of different origins, indicating a general cell response via a signaling process linked to the primary insult. In parallel to inhibition of Na,K-ATPase activity, expression of FXYD2 (either by transfection or induction) correlated with a reduction in rate of cell growth (Wetzel et al., 2004). An escape from cell growth delay was achieved by selective siRNA silencing of FXYD2 during on-going stress, suggesting the adaptive value of FXYD2 as a general cellular mechanism of regulation of cell growth.

Taken together, FXYD2 can be considered an endogenous inhibitory subunit of Na,K-ATPase affecting the pump's activity via reduction of affinity for Na^+^ and V_max_. The control occurs at multiple levels, such as alternative splicing, post-translational modification, and mRNA editing. The FXYD proteins evidently also participate in fine-tuning the signaling role of Na,K-ATPase.

## Functional properties of FXYD2 learned from knockout mice

Generation of knockout mice was an essential step toward characterization of FXYD2. Global knockout of FXYD2 was performed via replacement of the transmembrane domain coding sequence with a LacZ cassette with a selectable marker (Jones et al., 2005). The higher Na^+^ affinity in assays of kidney membranes from *Fxyd2*^−∕−^ knockout mice confirmed that FXYD2 normally reduces the Na^+^ affinity of Na,K-ATPase, thus complementing studies from transfected cells. The observed changes were in a physiological range suitable for modulation of Na,K-ATPase by hormones. No significant variation of K^+^ affinity or reduction in the affinity for ATP was observed between in *Fxyd2*^−∕−^ and WT, suggesting that alterations observed earlier in different expression systems (Therien et al., 1999; Arystarkhova et al., 2002a; Pu et al., 2002) might be cell specific.

## FXYD2^−∕−^ mice have a pancreatic phenotype

Although viable and fertile, the knockout mice had obvious problems with reproduction: reduced apparent conception rate, smaller surviving litter size, and reduced percentage of successful litters in pairs with *Fxyd2*^−∕−^ or even *Fxyd2*^+∕−^ dams (Arystarkhova et al., 2013). Thus deletion or even dose reduction of the *Fxyd2* gene was unfavorable for female reproduction. However, if pups survived the perinatal period (0–3 days), knockout mice lived a normal life-span with growth parameters not much different from wild type mice.

A renal phenotype was expected since kidney has the highest level of expression of FXYD2 (Arystarkhova et al., 2002a; Wetzel et al., 2004). However, initial analysis did not reveal any significant impairment in renal function in *Fxyd2*^−∕−^ mice kept under optimal care. Instead, a reduced blood glucose level was seen in *Fxyd2*^−∕−^ mice either under fed or fasted conditions (Arystarkhova et al., 2013). Although this could indicate peripheral resistance to insulin, either young or mature *Fxyd2*^−∕−^ mice revealed insulin sensitivity indistinguishable from their wild type controls. Remarkably, there was a dramatic improvement in glucose tolerance in the knockout animals. All of the above imply a metabolic phenotype of low blood glucose in *Fxyd2*^−∕−^ mice. Reduction in blood glucose correlated with an elevation in circulating insulin (2–2.5 fold) either under basal or glucose-stimulated conditions (Arystarkhova et al., 2013). Elevated insulin was sustained in pregnant *Fxyd2*^−∕−^ and *Fxyd2*^+∕−^ dams close to delivery (E18–E20) consistent with maternal pathophysiology as the reason for the fragility and low survival rate of newborn pups, by affecting either milk production or nurturing behavior.

Based on GEO profiles, pancreas is the second tissue (after kidney) in abundance of FXYD2 expression. By RT-PCR and Western blots, both splice variants of FXYD2 were identified in pancreatic islets from rodent and human tissues (Flamez et al., 2010). Immunofluorescence, however, revealed unusual localization of the protein considering its role as a regulatory subunit of Na,K-ATPase. While some of the FXYD2 was found at the plasma membrane (together with Na,K-ATPase), most FXYD2 was observed in the cytoplasm (Flamez et al., 2010; Arystarkhova et al., 2013). Whether the apparent separation of FXYD2 from Na,K-ATPase indicates intracellular trafficking of FXYD2 within beta cells in response to yet an unidentified physiological stimulus requires further investigation. We did not detect the edited mRNA product by RT-PCR. In pancreas FXYD2 may play a biological role distinct from regulation of Na,K-ATPase, and the molecular mechanism of intracellular retention may be interaction with another protein.

Functionally, there was no significant difference in ability to secret insulin from freshly isolated pancreatic islets from WT and *Fxyd2*^−∕−^ mice (Arystarkhova et al., 2013). However, morphometric analysis revealed a higher number of insulin-producing beta cells per islet cross-sectional area in the knockout mice compared to WT. This led to an increase in total beta cell mass/pancreas from the *Fxyd2*^−∕−^ knockouts (Arystarkhova et al., 2013). Thus depletion of FXYD2 from pancreatic islets apparently increases the rate of cell division, consistent with the effect of FXYD2 on growth rate in transfected cells in culture. Beta cell hyperplasia may underlie the elevation of insulin production that in turn may explain the observed phenotype of low blood glucose. No morphological changes were seen in acinar cells from *Fxyd2*^−∕−^ mice, implying preferential hyperplasia of the endocrine islet compartment. It should be noted that enhanced proliferation of beta cells in *Fxyd2*^−∕−^ mice stayed within the physiological range, i.e., unlike insulinomas. All together the data suggest that removal of FXYD2 is beneficial for pancreatic beta cells.

On the molecular level, freshly isolated islets from *Fxyd2*^−∕−^ mice exhibited significantly higher levels of phosphorylation of Akt (PKB; Arystarkhova et al., 2013), one of the central players in regulation of beta cell mass and function (Bernal-Mizrachi et al., 2001; Blandion-Rosano et al., 2012). This may underlie the islet hyperplasia observed in *Fxyd2*^−∕−^ mice, and reflect ongoing signaling in adults. Experimentally, inducible expression of FXYD2 in rat INS 832/13 cells was paralleled by a reduction in phosphorylation of Akt, whereas time-dependent degradation of FXYD2 resulted in its gradual elevation (Arystarkhova et al., 2013). PI3 kinase is the upstream activator of Akt kinase, and it had been shown to interact with Na,K-ATPase (Yudowski et al., 2000). A potential scenario is that depletion of FXYD2 leads to a conformational change in Na,K-ATPase and, as a result, sustained activation of PI3K, leading to enhanced activity of Akt. This would implicate FXYD2 as an upstream player in the Akt signaling pathway in pancreatic beta cells. Activation of Akt is one of the important links between growth signals and regulation of β-cell expansion (Bernal-Mizrachi et al., 2001; Elghazi et al., 2007). Downstream targets of Akt, tuberous sclerosis complex 1 and 2 (TSC1/2) and mechanistic target of rapamycin complex 1 (mTORC1), are prime candidates for enhancing cell cycle progression (Blandino-Rosano et al., 2012). The hope is that further investigation of FXYD2 will lead to novel regulators of beta cell mass to enhance insulin secretion for therapeutic purposes.

## Useful adaptations to FXYD2 depletion from kidney

Na,K-ATPase is located in basolateral membranes in renal tubules where it generates the driving force for Na^+^ entry across the apical membrane. Theoretically deletion of the inhibitory subunit FXYD2 should enhance renal Na^+^ reabsorption by increasing both Na^+^ affinity and V_max_ of Na,K-ATPase in the proximal tubule. Measured renal Na,K-ATPase activity in cortical membranes from the knockout mice was elevated as predicted (Arystarkhova et al., 2014), and so a renal phenotype was expected. Under basal conditions no significant differences between WT and knockout mouse were found in plasma concentration of Na^+^ or in plasma osmolality, implying no increase in sodium retention (Jones et al., 2005). Urine electrolytes (mg/mg creatinine) remained within the normal range in FXYD2-deficient mice (Jones et al., 2005). In addition, FXYD2-deficient mice were normotensive and demonstrated sodium balance indistinguishable from their littermate controls (Arystarkhova et al., 2014). Taken together, the experimental data imply that NaCl uptake or efflux in appropriate renal segments is adaptively controlled in the *Fxyd2*^−∕−^ mice.

Regulation of inflow through apical Na^+^ transporters is one possibility. Distal convoluted tubule (DCT) and thick ascending limb (TAL) are the segments with the highest level of expression of both Na,K-ATPase and FXYD2. The major apical Na^+^ transporters there are sodium/chloride cotransporter (NCC; Subramanya and Ellison, 2014) and sodium/potassium/chloride cotransporter (NKCC2; Ares et al., 2011) in DCT and cortical TAL (cTAL), respectively. Both belong to a family of electroneutral cation-coupled chloride co-transporters. Functionally, both transporters face the lumen of the tubules and perform uptake of Na^+^ from the tubular fluid driven by the sodium gradient created by Na,K-ATPase. Activity of both transporters is regulated by expression, trafficking and phosphorylation. In humans, reduced activity of NKCC2 leads to severe salt and volume loss and decreased blood pressure (Bartter syndrome), while reduction in NCC function is associated with salt wasting and low blood pressure (Gitelman syndrome). In contrast, gain of function of either transporter is associated with an increase of Na^+^ reabsorption and development of salt-sensitive hypertension (Ares et al., 2011; Hoorn et al., 2011).

If either TAL or DCT were involved in compensation of Na^+^ balance in the *Fxyd2*^−∕−^ mice, luminal sodium uptake should be reduced in those segments, i.e., activity of NKCC2 or NCC transporters should be diminished. Surprisingly, there was significant up-regulation of NCC protein expression (by 30%) and highly augmented phosphorylation of both cortical NKCC2 (at least two-fold) and NCC (4–6 fold at two phosphorylation sites, pT53 and pS71; Arystarkhova et al., 2014). The level of phosphorylation of both transporters is usually taken as a proxy of their activity. Thus high phosphorylation of NCC and NKCC2 in *Fxyd2*^−∕−^ mice would predict even higher Na^+^ reabsorption in cTAL and DCT that should eventually lead to hypertension. In practice, *Fxyd2*^−∕−^ mice revealed no significant difference in mean arterial pressure (either males or females) under basal conditions when compared to their wild type littermates (Arystarkhova et al., 2014). Thus the compensated physiology of the mouse and lack of Na^+^ retention cannot be explained by downregulation of apical Na^+^ entry in TAL and DCT segments.

Acute saline challenge of WT and *Fxyd2*^−∕−^ mice also did not reveal a difference in rate of Na^+^ excretion between genotypes (Arystarkhova et al., 2014). Therefore activation of both cortical NKCC2 and NCC must be explained by compensation in either more distal or more proximal segments in the knockout mice. No difference was seen in the expression level or activity of the distally-expressed amiloride sensitive epithelial sodium channel, ENaC (Arystarkhova et al., 2014), ruling it out for compensation for an increase in Na,K-ATPase activity, and leaving proximal tubules as a likely site of Na^+^ absorption adjustments in the *Fxyd2* knockout mouse.

A testable hypothesis is that increase in activity of Na,K-ATPase in proximal tubule, the principal site of Na^+^ reabsorption from filtrate, would cause excess reabsorption of Na^+^ into interstitial spaces and lead to activation of the intrarenal dopamine response (Aperia, 2000; Carey and Padia, 2013). This reduces the activity of apical NHE3 transporter, which is thought to be rate-limiting for Na^+^ reabsorption (McDonough, 2010), by trafficking it toward the base of the apical villi (Chen et al., 2009; McDonough, 2010), and internalizes Na,K-ATPase as well (Pedemonte et al., 2005; Gildea et al., 2009). The Na^+^ concentration in the lumen in proximal tubules would then stay high, and the observed hyperstimulation of the downstream NKCC2 and NCC transporters would be adaptive, bringing the FXYD2 knockout mouse into Na^+^ balance.

## Conclusions

To summarize, *Fxyd2*^−∕−^ mice are in a desirable state when considering the risk factors of metabolic syndrome. The animals are resistant to elevation of glucose in blood as well as to Na^+^ retention, i.e., they display features unfavorable for development of diabetes or salt-sensitive hypertension. In addition, deletion of FXYD2 from dorsal root ganglion neurons correlated with a marked reduction in a nociceptive adaptation (allodynia) to inflammation (Wang et al., 2015). Is there a common trait in these beneficial phenotypes observed in different tissues? We showed that FXYD2 modulates both ion transport (Arystarkhova et al., 1999, 2002a) and signaling (Arystarkhova et al., 2013). Investigation of mechanism(s) underlying favorable metabolic adaptations in the knockout mouse will be a challenge for the coming years.

## Author contributions

The author confirms being the sole contributor of this work and approved it for publication.

### Conflict of interest statement

The author declares that the research was conducted in the absence of any commercial or financial relationships that could be construed as a potential conflict of interest.
